# Progress in Medicine

**Published:** 1899-03-04

**Authors:** 


### Progress in Medicine.

### DISEASES OF THE HEART AND CIRCULATION.

**The Pulse.**—Oliver,[1] in a lecture on the pulse and blood pressure, says that though it is possible to measure the external diameter of the radial artery by the finger, the bore of the vessel cannot be so measured. This can, however, be done by the arteriometer, the use of which shows that it is lessened in atheroma, syphilis, and chronic gout, whilst it is enlarged when the blood pressure is considerably above the normal. The blood pressure can be accurately measured by the hæmadynometer. Observations with this instrument show that the arterial pressure is 10 to 15 mm. higher in the sitting or standing position than in recumbency. It is raised by muscular exercise, by mental exercise, especially if accompanied by emotional excitement. Rest and sleep lower the blood pressure to its lowest point. The mere taking of fluids or food produces a rise of blood pressure before the act of digestion sets in. This is the explanation of the usefulness of getting a fainting person to swallow something; warmth relaxes the arterioles, and thus lessens the arterial pressure, whilst cold has the opposite effect. The pulse rate is also of importance; if it is considerably raised in frequency the pressure rises. Thus all the physiological causes of variation, except sleep and rest, raise the blood pressure.

The average arterial blood pressure is about 100 mm. of mercury during recumbency, and 110 or 115 mm. in sitting or standing posture. In clinical observation it is the increase, not the decrease, of blood pressure which is of significance. This is nearly always due primarily to a peripheral cause, which is either organic changes in the peripheral vessels or contraction of the arterioles. Heightened arterial pressure is, as is well known, apt to lead to dilatation of the heart, also to disturbance of the breathing, especially on movement, and, thirdly, to dilatation of the arterioles. It is found that when the arterial pressure is high there is an increase in the calibre of the vessel. The leading signs of increased arterial blood pressure are the following: (1) The artery is felt to be full, and can often be rolled under the finger. (2) The beat of the pulse is prolonged. (3) The pulse is only obliterated under increased pressure. (4) The hæmadynometer defines the amount of this pressure with accuracy. (5) The blood pressure, arterial and venous, is frequently uniform or alters but little on changes of posture, and the radial pulse is well sustained when the wrist is elevated above the head. (6) The second aortic sound is generally accentuated. (7) The apex beat is frequently moved to the left of the normal position. (8) The breathing is easily disturbed on slight exertion.

Mackensie[2] has for some years made observations on the alleged retardation of the pulse in aortic regurgitation. It is held by Balfour and Broadbent that the pulse wave takes longer to reach the radial and other distal arteries in this disease than it does in health. Mackensie has made accurate measurements by recording simultaneously the apex beat, the carotid and the radial pulses. The tracings show that there is no more retardation of the pulse in aortic regurgitation than in health.

**Functional Hypertrophy of the Arteries.**—Morkstun[3] from numerous experiments concludes that, under the influence of gymnastic exercises, not only do the muscles undergo hypertrophy, but the arteries which nourish them also increase in size. This enlargement occurs in the transverse as well as in the longitudinal diameter. It is very likely that a functional hypertrophy of the arteries in all the other organs may occur as soon as they are called upon to increase their functions. This is a physiological process which renders the work of the organ somewhat easier. On the other hand, greatly hypertrophied arteries form a fertile soil for the development of sclerosis.

**Obliteration of the Aorta.**—Brunner[4] has recently recorded an instance of this rare deformity. The patient, a man aged 30, came under observation for, and died from phthisis. The condition was approximately diagnosed during life from the enlarged and tortuous arteries in the upper part of the thorax. Post-mortem, the aorta was found closed by an oblique membrane below the insertion of the ductus arteriosus. This is in accord with cases already recorded. The stenosis is always situated at the level of, or just above or below, the ductus arteriosus, which in most cases is itself obliterated. As a rule there is good collateral circulation through the branches going to the trunk from the subclavian arteries, and little or no discomfort has been experienced. These cases probably arise from an abnormal prolongation into the aortic wall of the tissues which go to form the wall of the ductus arteriosus. It is probably gradually developed during the first days of independen existence.

**Tuberculous Endocarditis and Arteritis.**—It was thought at one time that endocarditis and tuberculosis were mutually exclusive. But morbid anatomy has, demonstrated that the two lesions may occur together. Thus, whilst in the majority of cases of phthisis associated with endocarditis, streptococci, and staphylococci are the commonest forms found, in some cases tubercle bacilli have also been discovered in the vegetations. V. Leyden, for instance, found tubercle bacilli in three consecutive cases of phthisis associated with endocarditis. Michaelis and Blum[5] have recently made experiments on the question by setting up aortic incompetence in rabbits by piercing the valves with an instrument introduced through the carotid, and subsequently injecting tubercle bacilli into the circulation. A diffuse tuberculosis was set up, and in the copious vegetations which had formed on the aortic valves the tubercle bacillus was demonstrated.

Blumer[6] has recorded two cases of tuberculosis of the aorta. It was shown long ago by Weigert and by Koch that the walls of small veins and arteries may become the seat of tubercle in general tuberculosis, but instances in which the larger arteries are implicated are very rare. Of these latter cases some have been due to direct extension from a tuberculous process originating outside the vessel, whilst in others —*e.g.*, the cases here reported—the tubercle was probably directly implanted on the vessel wall. The lesions take the form of nodules springing from the intima and projecting into the lumen of the aorta or other vessel. The bacilli might be carried to the inner coat of the vessel through the vasa vasorum, but this is not probable, because the middle coat shows no changes, and in the aorta, as in the cases described, the vasa vasorum do not penetrate to the inner coat, which is the site of the lesion. The bacilli then probably are directly implanted from the blood stream, exactly as occurs in some forms of endocarditis. It is presumable that a previous lesion of the intima would favour the development of tubercles, but in the cases under discussion there was no indication that such changes were present. It is conceivable that in cases of this sort, where large quantities of bacterial toxins are circulating in the blood stream, these may cause localised areas of lessened resistance in the vessel wall, and so prepare it for the lodgement of the tubercle bacilli.

**Cardio-pulmonary Murmur.**—Squire[7] has recorded a good many cases of a respiratory murmur rhythmical with the heart beats. It is well known that consolidation of the lung conducts sound from the heart or large vessels to the surface of the chest, *e.g.*, in phthisis. Accordingly if we hear sounds corresponding in rhythm to the beat of the heart at some distance from that organ, we are led to suspect that there is consolidation of the intervening lung. But there is a fallacy. There may be heard sometimes a bruit or whiff, which is caused by movement of the air in the bronchial tubes and lungs; due to and synchronous with the beat of the heart, and not due to cardiac disease or consolidation of the lung. The murmur is only the ordinary respiratory murmur momentarily modified in intensity at the systole of the heart. There is little significance in the sound *per se*, though it is always associated with general debility. Its importance lies in the risk of mistaking it for a sign of disease of the heart valves, and of suspecting some lung mischief from the apparent conduction of a cardiac murmur to a distant part.

**Morphia in Cardiac Disease.**—Toogood[8] recommends the administration of morphia in those distressing cases of cardiac disease (mainly of mitral incompetence), where the digitalis and its allies, strophanthus and convallaria, appears to do nothing more than excite persistent vomiting, and where the heart is extremely irritable and irregular in rhythm and the pulse in volume, where often an ever-present dyspnœa renders the condition of the patient intolerable from exhaustion and want of sleep, and where there may be also œdema from a failing circulation, and a scanty amount of albuminous urine. In these cases the hypodermic injection of morphia often effects the most gratifying results; the pulse becomes steady, strong, and regular, the œdema disappears, the dyspnœa is relieved, and the urine becomes normal in amount and character, and the albumen becomes much less or entirely disappears. Quarter-grain may be injected twice or thrice in the 24 hours.

**Pericarditis.**—Brentano[9] is of opinion that operative interference is indicated only in exudative pericarditis, and even then only when the life of the patient is threatened or a purulent inflammation is suspected. There is no point at which puncture can be made with positive safety to the heart, for when the pericardial sac is distended with fluid the heart takes up a position against the anterior chest wall, unless held in some other position by adhesions. Brentano hence advises incision of the pericardial sac after resection of a rib. This can be done under local anæsthesia only. The fifth left cortal cartilage is resected, the internal mammary arteries then doubly tied and divided, and an incision then made in the glistening whitish pericardial membrane. The fluid escapes in spurts, because the heart shows a tendency to close the opening. In purulent exudation irrigation with sterilised water is recommended. The incised edges of the pericardium should be sutured to the skin and the cavity drained by strips of iodoform gauze.

[1] Clinical Jour., Feb. 1, 1899. [2] Edinburgh Med. Jour., Oct., 1898. [3] Wratch 39, 1898. [4] Corrend. Blatt Schwertz Aertz, Jan. 1, 1899. [5] Deut. Med. Woch, Sept. 1, 1898. [6] Amer. Jour. Med. Sci., Jan., 1899. [7] Brit. Med. Jour., Dec. 10, 1898. [8] Lancet, Nov. 26, 1898. [9] Deut. Med. Woch, No. 82., 1898.

